# Novel Anticancer Fused Pyrazole Derivatives as EGFR and VEGFR-2 Dual TK Inhibitors

**DOI:** 10.3389/fchem.2019.00917

**Published:** 2020-01-24

**Authors:** Nashwa M. Saleh, Marwa G. El-Gazzar, Hala M. Aly, Rana A. Othman

**Affiliations:** ^1^Department of Chemistry, Faculty of Science (Girl's), Al-Azhar University, Cairo, Egypt; ^2^Department of Drug Radiation Research, National Center for Radiation Research and Technology, Egyptian Atomic Energy Authority, Cairo, Egypt

**Keywords:** fused pyrazoles, anticancer, HEPG2, EGFR, VEGFR-2

## Abstract

EGFR and VEGFR-2 represent promising targets for cancer treatment as they are very important in tumor development as well as in angiogenesis and metastasis. In this work, 6-amino-4-(2-bromophenyl)-3-methyl-1,4-dihydropyrano[2,3-c]pyrazole-5-carbonitrile **1** and (E)-4-(2-Bromobenzylidene)-5-methyl-2,4-dihydro-3H-pyrazol-3-one **11** were selected as starting materials to synthesize different fused pyrazole derivatives; dihydropyrano[2,3-c]pyrazole **1**, **2**, **7**–**9**, and **15**, pyrazolo[4′,3′:5,6]pyrano[2,3-d]pyrimidine **3–6**, pyrazolo[3,4-d]pyrimidine **12** and **13**, and pyrazolo[3,4-c]pyrazole **14** derivatives were synthesized to evaluate their anticancer activity against HEPG2 human cancer cell lines compared to erlotinib and sorafenib as reference drugs. Seven compounds **1**, **2**, **4**, **8**, **11**, **12**, and **15** showed nearly 10 fold higher activity than erlotinib (10.6 μM) with IC_50_ ranging from 0.31 to 0.71 μM. *In vitro* EGFR and VEGFR-2 inhibitory activity were performed for the synthesized compounds, and the results identified compound **3** as the most potent EGFR inhibitor (IC_50_ = 0.06 μM) and compound **9** as the most potent VEGFR-2 inhibitor (IC_50_ = 0.22 μM). Moreover, compounds **9** and **12** revealed potent dual EGFR and VEGFR-2 inhibition, and these results were supported by docking studies of these two compounds within the active sites of both enzymes.

## Introduction

Cancer is considered a worldwide health risk nowadays. It is the second driving cause of death in the world, and it is anticipated to be the primary cause of death in the upcoming years (Harding et al., 2018). The invention of novel small molecules that are both potent and selective antitumor agents continues to be a serious challenge to medicinal chemistry researchers (Zhong et al., 2014; Iyer, 2015; Lin et al., 2019). In spite of the indispensable advances accomplished over the last decades in the design and development of assorted anticancer agents, current accessible treatments still have two significant limitations, the primary being the shortage of selectivity for cancer tissues (Shafei et al., 2017), inflicting unwanted side effects (Cavero-Redondo et al., 2015; Brewer et al., 2016; Decalf et al., 2017). The second is the acquisition of multiple-drug resistance by cancer cells, rendering them unresponsive to standard therapy (Wu et al., 2014). Unwanted side effects of anticancer medication can be overcome with agents capable of discriminating tumor cells from normal cells. Hepatocellular carcinoma is taken into account because it is the third most common cancer in males, the seventh in females, and the third most prevalent cause of mortality associated with cancer round the world, and this is why early diagnosis is of great importance (Wen et al., 2019).

The inhibition of the epidermal growth factor receptor (EGFR) and vascular endothelial growth factor receptor (VEGFR-2) plays a crucial role in tumor suppression, angiogenesis, and metastasis (Alferez et al., 2008; Mghwary et al., 2019). The two share common signaling pathways. EGFR inhibition can diminish VEGF expression and attenuate angiogenesis while also prompting VEGFR-2 upregulation, eventually to a resistance equal to that of EGFR inhibitors (Lang et al., 2008). Consequently, dual inhibition of EGFR and VEGFR-2 became helpful for targeting and treating cancer by acting synergistically (Xi et al., 2013). Both enzymes share in their structural features the presence of an ATP binding site, permitting several small molecules to bind and act as dual inhibitors as gefitinib (AstraZeneca, London, UK, 2003), erlotinib (Genentech, South San Francisco, CA, USA and OSIP, Melville, NY, USA, 2004), sorafenib (Bayer, Leverkusen, Germany and Onyx, South San Francisco, CA, USA, 2005), vandetanib (Bristol-Myers Squibb, New York, NY, USA, 2006), lapatinib (GlaxoSmithkline, London, UK, 2007), regorafenib (Bayer, Leverkusen, 2012), and afatinib (Boehringer Ingelheim, Ingelheim, Germany, 2013). Most of these small molecules are quinazoline-based compounds, and many attempts have been made to their structural modification to produce potent anticancer agents. The quest for new scaffolds able to act as tyrosine kinase inhibitors has thus an ongoing challenge nowadays (Breen and Soellner, 2015; Fabbro, 2015; Berndt et al., 2017; Fischer, 2017).

**Figure 1:**
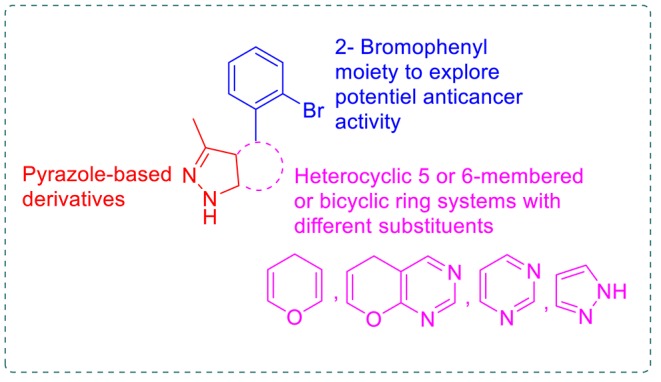
The designed pyrazole-based target compounds.

Pyrazole and fused pyrazole systems, such as pyranopyrazole and pyrazolpyrimidines, are promising scaffolds for many anticancer agents (Chauhan and Kumar, 2013; Ansari et al., 2017; Shukla et al., 2019). Moreover, many brominated marine natural products, such as eudistomin alkaloid, exhibited anticancer activity (Rajesh and Annappan, 2015; Florean et al., 2016). In addition to many synthetic brominated compounds (Ökten et al., 2017). Based on these facts and in continuation of previous work aimed at developing new anticancer agents (Aly, 2009, 2011; Ghorab et al., 2010, 2016, 2018; Aly and El-Gazzar, 2012; Aly and Kamal, 2012; El-Gazzar and Aly, 2018; El-Gazzar et al., 2018, 2019), novel pyrazole-based derivatives with 2-bromophenyl moiety were synthesized by fusing different heterocyclic rings to the pyrazole in order to study the resulting anticancer effects (Figure 1). So, novel dihydropyrano[**2,3**-c]pyrazole **1**, **2**, **7–9**, and **15**, pyrazolo[**4**′, **3**′:**5,6**]pyrano[**2,3**-d]pyrimidine **3-6**, pyrazolo[**3,4**-d]pyrimidine **12** and **13**, and pyrazolo[**3,4**-c]pyrazole **14** derivatives were synthesized to evaluate their antitumor activity against the HEPG2 human cancer cell line; compared to erlotinib and sorafenib as reference drugs, the *in vitro* EGFR and VEGFR-2 inhibitory activity and the docking mode of the most potent candidates were evaluated to explain the obtained inhibitory activity.

## Materials and Methods

All chemicals used in this study were of analytical reagent grade and of the highest purity available. Organic solvents were purchased from British Drug House (BDH). Melting points (°C, uncorrected) were determined in the open capillaries on a Gallenkemp melting point apparatus (Sanyo Gallenkemp, Southborough, UK). Precoated silica gel plates (silica gel 0.25 mm, 60 GF 254; Merck, Germany) were used for thin layer chromatography, dichloromethane/methanol (9.5:0.5 mL) mixture was used as a developing solvent system at room temperature, and the spots were visualized by ultraviolet light and/or iodine. Microanalytical determinations (C, H, and N) were carried out on Carlo Erba 1108 Elemental Analyzer (Heraeus, Hanau, Germany) and were within ±0.4 of the calculated values. The IR spectra were recorded on a Perkin-Elmer 437 IR spectrophotometer (400–4,000 cm^−1^) (KBr technique) (Waltham, Massachusetts, USA). NMR spectra (DMSO-*d*_6_) were recorded on a Bruker spectrophotometer (400 MHz for ^1^H NMR and 100 MHz for ^13^C NMR) (Bremen, Germany).

### Chemistry

#### Ethyl(E)-N-(4-(2-bromophenyl)-5-cyano-3-methyl-1,4-dihydropyrano[2,3-c pyrazol-6-yl)formimidate (2)

A solution of compound **1** (Muramulla and Zhao, 2011) (0.5 g, 0.0015 mol) in triethylorthoformate (10 ml) containing three drops of acetic anhydride was refluxed for 8 h. The reaction mixture was cooled and poured onto ice water, and the obtained solid was recrystallized from methanol to produce white crystals **2**: yield, 98%; m.p. 158–160°C; IR, cm^−1^: 3,154 (CH arom.), 2,190 (C≡N), 1,600 (C=N). ^1^H NMR (DMSO-*d*_6_*)* δ_*H*_: 1.32 (t, 3H, CH_3_ ethyl), 2.10 (s, 3H, CH_3_ pyrazole), 4.34 (q, 2H, CH_2_), 5.36 (s, 1H, pyrane-H), 7.26–7.68 (m, 5H, Ar-H+NH, D_2_O exch.), 8.67 (s, 1H, N=CH). ^13^C NMR (DMSO-*d*_6_) δ_*C*_: 13.26 (CH_3_ pyrazole), 14.27 (CH_3_ ethyl), 23.49 (CH), 64.76 (CH_2_), 80.66 (C-CN), 117.34 (CN), 105.10, 123.11, 129.16, 130.47, 131.90, 133.59, 140.70, 156.17, 162.32, 171.23, 158.43 (N=CH). MS, m/z (%): (387) (M^+^) (45), (386.9) (100). Anal. Calcd. For C_17_H_15_BrN_4_O_2_ (387): C, 52.73%; H, 3.90%; N, 14.47%. Found: C, 52.92%; H, 4.20%; N, 14.09%.

#### 4-(2-Bromophenyl)-5-imino-3-methyl-1,4-dihydropyrazolo[4′,3′:5,6]pyrano[2,3-d]pyrimidin-6(5H)-amine (3)

A mixture of **2** (0.39 g, 0.001 mol), absolute ethanol (15 ml), and 80% hydrazine mono-hydrate (0.05 ml, 0.001 mol) was refluxed for 8 h. The formed precipitate was filtered, dried, and crystallized from ethanol to produce golden crystals **3**: yield; 38% m.p. 120–122°C; IR, cm^−1^: 3,421, 3,236 (NH_2_/2NH), 2,997 (CH arom.), 2,850 (CH aliph.), 1,612 (C=N). ^1^H-NMR (DMSO-*d*_6_*)* δ_*H*_: 2.03 (s, 3H, CH_3_), 4.08 (s, 1H, CH), 7.50–8.94 (m, 7H, Ar-H+ NH_2_, D_2_O exch.), 11.21, 11.22 (br, 2H, 2NH, D_2_O exch.). ^13^C NMR (DMSO-d6) δC: 10.44 (CH_3_), 28.26 (CH), 102.25 (C=C), 124.11, 128.00, 128.24, 128.62, 130.38, 130.46, 131.86, 132.61, 133.27, 136.83, 161.59. MS, m/z (%): (373) (M^+^) (4.48), (205) (100); Anal. Calcd. For C_15_H_13_BrN_6_O (373): C, 48.27%; H, 3.51%; N, 22.52%. Found: C, 47.89%; H, 3.31%; N, 22.73%.

#### 4-(2-Bromophenyl)-3,7-dimethyl-4,6-dihydropyrazolo[4′,3′:5,6]pyrano[2,3-d]pyrimidin-5(1H)-one (4)

A solution of compound **1** (Muramulla and Zhao, 2011) (0.5 g, 0.0015 mol) in acetic anhydride (20 ml) was refluxed for 10 h, the reaction mixture was then concentrated, and the separated solid was recrystallized from ethanol to produce a white powder **4**: yield, 40%; m.p. 115°C; IR, cm^−1^: 3,178 (NH), 3,062 (CH arom.), 2,927 (CH aliph.), 1,708 (C=O), 1,589 (C=N). ^1^H NMR (DMSO*d*_6_) δ_*H*_: 1.88 (s, 3H, CH_3_), 2.26 (s, 3H, CH_3_ pyrimidine), 5.33 (CH), 7.04–7.51 (m, 4H, Ar-H), 12.10, 12.33 (s, 2H, 2NH D_2_O exch.).^13^C NMR (DMSO*d*_6_) δ_*C*_: 11.05 (CH_3_ pyrazole), 21.53 (CH_3_ pyrimidine), 36.06 (CH), 101.15 (C=C), 123.69, 128.05, 128.39, 130.10, 132.87, 136.31, 137.33, 143.59, 144.05, 172.49, 173.13, 158.99 (N=C-NH), 160.05 (C=O). Anal. Calcd. For C_16_H_13_BrN_4_O_2_ (373): C, 51.49%; H, 3.51%; N, 15.01%. Found: C, 51.21%; H, 3.83%; N, 14.91%.

#### 4-(2-Bromophenyl)-3-methyl-4,6-dihydropyrazolo [4′,3′:5,6]pyrano[2,3-d]pyrimidin-5(1H)-one (5)

A solution of compound **1** (Muramulla and Zhao, 2011) (0.5 g, 0.0015 mol) in formic acid (20 mL) was refluxed for 5 h. The reaction mixture was cooled and then poured onto cold water, and the obtained solid was crystallized from ethanol to produce white crystals **5**: yield, 79%; m.p. 205°C; IR, cm^−1^: 3,064 (CH arom.), 2,925 (CH aliph.), 1,715 (C=O), 1,585 (C=N). ^1^H NMR (DMSO-*d*_6_*)* δ_*H*_: 2.05 (s, 3H, CH_3_), 4.53 (s, 1H, CH), 7.05–7.59 (m, 6H, Ar-H+ NH,D_2_O exch.), 10.96 (s, 1H, NH, D_2_O exch.). ^13^C NMR (DMSO-d6) δC: 10.95 (CH_3_), 36.07 (CH), 101.64 (C=C), 120.25, 123.71, 128.07, 128.44, 130.08, 132.91, 137.61, 143.50, 151.64, 157.09, 160.25, 173.17(C=O). MS, m/z (%): (358) (M-1) (11.73), (126) (100). Anal.Calcd. For C_15_H_11_BrN_4_O_2_ (359): C, 50.16%; H, 3.09%; N, 15.60%. Found: C, 50.43%; H, 2.88%; N, 15.34%.

#### 4-(2-Bromophenyl)-3-methyl-1,4-dihydropyrazolo[4′,3′:5,6]pyrano[2,3-d]pyrimidin-5-amine (6)

A solution of compound **1** (Muramulla and Zhao, 2011) (0.5 g, 0.0015 mol) in formamide (20 mL) was refluxed for 4 h. The reaction mixture was cooled and then poured onto cold water, and the obtained solid was crystallized from dioxane to produce a compound **6**: yield, 84%; m.p. 320°C; IR, cm^−1^: 3,390, 3,232 (br., NH_2_\NH), 2,924 (CH arom.), 2,893 (CH aliph.), 1,608 (C=N). ^1^H NMR (DMSO-*d*_6_*)* δ_*H*_: 1.87 (s, 3H, CH_3_), 4.41 (s, 1H, CH), 7.14(s, 2H, NH_2_, D_2_O exch.), 7.32–9.10 (m, 6H, Ar-H+ NH, D_2_O exch.). ^13^C NMR (DMSO-d6) δC: 11.13(CH_3_), 33.68 (CH), 103.70 (C=C), 123.00, 123.17, 124.92, 127.57, 128.86, 130.85, 132.28, 133.49, 135.42, 147.27, 160.64. MS, m/z (%): (357) (M-1) (54), (68) (100). Anal. Calcd. For C_15_H_12_BrN_5_O (358): C, 50.30%; H, 3.38%; N, 19.55%. Found: C, 49.98%; H, 3.74%; N, 19.23%.

#### N-(4-(2-Bromophenyl)-5-cyano-3-methyl-1,4-dihydropyrano[2,3-c]pyrazol-6-yl)-2-cyanoacetamide (7)

A mixture of compound **1** (Muramulla and Zhao, 2011) (0.5 g, 0.0015 mol) and ethyl cyanoacetate (10 mL) was refluxed for 5 h. The formed solid was collected and crystallized from dioxane to produce a black powder **7**: yield, 98%; m.p. > 360°C; IR, cm^−1^: 3,208 (NH), 2,937 (CH arom.), 2,199 (C≡N), 1,640 (C=O), 1,536 (C=N). ^1^H NMR (DMSO-*d*_6_) δ_*H*_: 1.88(s, 3H, CH_3_), 4.08 (s, 2H, CH_2_), 5.40 (s, 1H, CH pyrane), 7.17–7.96 (m, 4H, Ar-H), 11.60, 11.64 (2s, 2H, 2NH, D_2_O exch.).^13^C NMR (DMSO-*d*_6_*)*δ_*C*_: 14.43 (CH_3_), 24.95 (CH_2_-CN), 25.07(CH),62.39 (C-CN),115.56, 116.04 (2CN), 124.86, 127.19, 133.20, 135.86, 138.86, 141.70, 145.36, 151.07, 155.41, 164.78,166.18 (C=O). MS, m/z (%): (M^+^) (398) (20.42), (399) (M+1) (2.08), (103) (100). Anal Calcd. For C_17_H_12_BrN_5_O_2_ (398): C, 51.27%; H, 3.04%; N, 17.59%. Found: C, 50.99%; H, 3.38%; N, 17.88%.

#### 1-(4-(2-Bromophenyl)-5-cyano-3-methyl-1,4-dihydropyrano[2,3-c]pyrazol-6-yl)-3-phenylthiourea (8)

A mixture of compound **1** (Muramulla and Zhao, 2011) (0.5 g, 0.0015 mol) and phenyl isothiocyanate (0.2 mL, 0.0015 mol) in absolute ethanol (30 mL) was refluxed for 6 h. The reaction was cooled and the obtained solid was crystallized from dioxane to produce a yellow powder **8**. Yield: 80%; m.p.: 230°C; IR, cm^−1^: 3,452 (br, NH), 2,935 (CH arom.), 2,854 (CH aliph.), 2,206 (C≡N), 1,627 (C=N), 1,442 (C=S). ^1^H NMR (DMSO*d*_6_) δ_*H*_: 1.77 (s, 3H, CH_3_), 5.08 (s, 1H, CH), 6.96–7.59 (m, 9H, Ar-H), 12.14 (s, 3H, 3NH). ^13^C NMR (DMSO*d*_6_) δ_*C*_: 10.14 (CH_3_), 36.32 (CH), 56.48 (C-CN), 97.52 (CN), 120.79, 122.90, 128.86, 129.37, 131. 42, 133.09, 135.89, 143.07, 155.37, 158.37, 160.02, 161.69, 172.49, 173.13, 176.01 (C=S). Anal. Calcd. For C_21_H_16_BrN_5_OS (466): C, 54.09%; H, 3.46%; N, 15.02%. Found: C, 53.88%; H, 3.75%; N, 15.31%.

#### N-(4-(2-Bromophenyl)-5-cyano-3-methyl-1,4-dihydropyrano[2,3-c]pyrazol-6-yl)-4-methyl-benzenesulfonamide (9)

A mixture of compound **1** (Muramulla and Zhao, 2011) (0.5 g, 0.0015 mol) toluene sulfonyl chloride (0.28 g, 0.0015 mol) in benzene (20 mL) containing 3 drops of pyridine was refluxed for 8 h. The reaction mixture was concentrated and then acidified with diluted HCl. The solid obtained was crystallized from methanol to produce a brown powder **9**. Yield, 50%; m.p. 193–195°C; IR, cm^−1^: 3,267 (NH), 3,155 (CH arom.), 2,873, 2,843 (CH aliph.), 2,191 (C≡N), 1,612 (C=N), 1,384, 1,165 (SO_2_). ^13^C NMR (DMSO-*d*_6_*)*δ_*C*_: 16.05,16.20 (2CH_3_), 31.26 (CH), 57.50 (C-CN), 118.91 (CN), 139.29 (C-SO_2_), 120.78, 123.54, 126.00, 128.06, 128.61, 130.62, 131.66, 146.36, 161.92, 162.83, 165.45, 167.19, 167.77.MS, m/z (%): (485) (M^+^) (24.61), (487) (M+2) (21), (247) (100). Anal Calcd. For C_21_H_17_BrN_4_O_3_S (485): C, 51.97%; H, 3.53%; N, 11.54%. Found: C, 52.07%; H, 3.88%; N, 11.31%.

#### (E)-4-(2-Bromobenzylidene)-5-methyl-2,4-dihydro-3H-pyrazol-3-one (11)

A mixture of **10** (Yusuf et al., 2017) (0.5 g, 0.005 mol), 2-bromobenzaldehyde (0.9 mL, 0.01 mol) in ethanol (15 mL) in presence of piperidine (1 mL) was refluxed for 5 h. The precipitated solid filtered off, washed with ethanol, dried, and crystallized from ethanol to produce an orange powder **11**. Yield, 90.3%; m.p. 210°C; IR, cm^−1^: 3,224 (NH), 3,062 (CH arom.), 2,862 (CH aliph.), 1,658 (C=O), 1,612 (C=N). ^1^H NMR (DMSO-*d*_6_*)* δ_*H*_: 1.83 (s, 3H, CH_3_), 8.57 (s, 1H, CH), 6.48–7.09 (m, 4H, Ar-H), 11.28 (s, 1H, NH, D_2_O exch.). ^13^C NMR (DMSO-*d*_6_*)* δ_*C*_: 11.07 (CH_3_), 128.02 (CH=CH), 128.26, 128.93, 130.59, 131.39, 132.82, 134.49, 172.49, 192.20 (C=O). Anal. Calcd. For C_11_H_9_BrN_2_O (265): C, 49.84%; H, 3.42%; N, 10.57%. Found: C, 50.07%; H, 3.56%; N, 10.44%.

#### 4-(2-bromophenyl)-3-methyl-1, 3a, 4, 5-tetrahydro-6h-pyrazolo [3,4-d]pyrimidin-6-one (12)

A mixture of **11** (0.47 g, 0.0018 mol) and urea (0.1 g, 0.0018 mol) in glacial acetic acid (10 mL) was refluxed for 5 h. The solid product precipitated after cooling was filtered off, dried, and crystallized from ethanol to produce a deep orange powder **12**. Yield, 49%; m.p. 205°C; IR, cm^−1^: 3,228 (NH), 3,059 (CH arom.), 2,893 (CH aliph.), 1,650(C=O), 1,612 (C=N). ^1^H NMR (DMSO-*d*_6_*)* δ_*H*_: 1.98 (s, 3H, CH_3_), 5.06 (d, 1H, pyrimidine-H), 7.09–7.87 (m, 5H, Ar-H), 10.96, 11.06 (br, 2H, 2NH, D_2_O exch.). ^13^C NMR (DMSO-d6) δC: 11.06 (CH_3_), 31.25 (CH Pyrimidinone), 55.37 (CH), 128.93, 129.61, 130.59, 131.37, 133.11, 133.63, 133.92, 161.18, 192.20 (C=O). MS, m/z (%): (292) (M-CH_3_) (21), (185) (100). Anal. Calcd. For C_12_H_11_BrN_4_O (307): C, 46.93%; H, 3.61%; N, 18.24%. Found: C, 46.67%; H, 3.88%; N, 17.99%.

#### 4-(2-Bromophenyl)-3-methyl-1,3a,4,5-tetrahydro-6H-pyrazolo[3,4-d]pyrimidine-6-thione (13)

A mixture of **11** (0.47 g, 0.0018 mol) and thiourea (0.13 g, 0.0018 mol) in glacial acetic acid (10 mL) was refluxed for 5 h. The solid product separated after cooling was filtered off, dried, and crystallized from ethanol to produce a brown powder **13** Yield, 58%; m.p. 170°C; IR, cm^−1^: 3,202 (NH), 3,041 (CH arom.), 2,843 (CH aliph.), 1,611 (C=N), 1,406, 1,023 (2C=S). ^13^C NMR (DMSO-*d*_6_*)* δ_*C*_: 15.17 (CH_3_), 45.18 (CH), 46.68 (CH), 121.19, 127.77, 129.53, 133.30, 153.37, 167.87, 184.87 (C=S). MS, m/z (%): (323) (M^+^) (28.44), (325) (M^+^+2) (14.07), (185) (100). Anal. Calcd. For C_12_H_11_BrN_4_S (323): C, 44.59%; H, 3.43%; N, 17.33%. Found: C, 44.22%; H, 3.71%; N, 17.06%.

#### 4-(2-Bromophenyl)-3-methyl-1,3a,4,5-tetrahydropyrazolo[3,4-c]pyrazole (14)

A mixture of **11** (0.47 g, 0.0018 mol), hydrazine monohydrate (0.09 mL, 0.0018 mol) in ethanol (20 mL) was refluxed for 5 h. The solid product separated after cooling was filtered off, dried, and crystallized from ethanol to produce a compound **14**. Yield, 27%; m.p. 140°C; IR, cm^−1^: 2,992 (CH arom.), 1,603(C=N). ^1^H NMR (DMSO-*d*_6_*)* δ_*H*_: 1.74 (s, 3H, CH_3_), 1.83 (s, 1H, CH), 3.66 (br,1H, CH-Ar), 7.05–8.94 (m, 6H, Ar-H+2NH). ^13^C NMR (DMSO-d6) δC: 11.08 (CH_3_), 34.35 (CH), 44.06 (CH), 124.07, 127.58, 128.77, 130.34, 135.68, 136.67, 139.71, 161.19. Anal. Calcd. For C_11_H_11_BrN_4_ (279): C, 47.33%; H, 3.97%; N, 20.07%. Found: C, 47.19%; H, 4.07%; N, 20.24%.

#### 4-(2-Bromophenyl)-3-methyl-6-oxo-1,6-dihydropyrano[2,3-c]pyrazole-5-carbonitrile (15)

A mixture of **11** (0.47 g, 0.0018 mol), and ethylcyanoacetate (0.2 mL, 0.0018 mol) in sodium ethoxide (0.5 g in 20 mL) was refluxed for 5 h. The reaction mixture was poured onto ice water, the precipitated solid was filtered off, dried, and crystallized from ethanol to produce compound **15**. Yield, 66%; m.p. >360°C; IR, cm^−1^: 3,373 (NH), 3,056 (CH arom.), 2,922 (CH aliph.), 2,198 (C≡N), 1,694 (C=O), 1,594 (C=N). ^1^H NMR (DMSO-d6) δH: 2.51(s, 3H, CH_3_), 3.89 (S, 1H, NH, D_2_O exch.), 7.20–7.80 (m, 4H, Ar-H).^13^C NMR (DMSO-*d*_6_*)* δ_*C*_: 14.17 (CH_3_), 56.49 (C-CN), 117.19 (CN), 128.02, 128.26, 128.93, 130.6, 131.4, 132.8, 134.5, 136.5, 158.03 (9 C, 172.04 (C=O). MS, m/z (%): (330) (M^+^) (22.39), (298) (100). Anal. Calcd. For C_14_H_8_BrN_3_O_2_ (330): C, 50.93%; H, 2.44%; N, 12.73%. Found: C, 51.21%; H, 2.63%; N, 12.91%.

### *In vitro* Biological Evaluation

#### *In vitro* Anticancer Activity

The MTT method of monitoring *in vitro* cytotoxicity was used with multiwell plates. The stock concentration of the entire synthesized compounds in DMSO was 10 mM, and this was used to prepare the working dilution. The final DMSO concentration used in the experiments was ≤ 0.5% as the working concentration. Human liver cancer cell lines (HepG2) were cultured according to the manufacturer's instructions. The compounds in serial dilutions (0.01, 0.1, 1.0, 10, and 100 μM) were added after 24 h of culture and the cells were cultured for another 24 h at 37°C. The cell viability was determined in each experiment using MTT (3-(4, 5-dimethylthiazol-2-yl)-2, 5-diphenyltetrazolium bromide) colorimetric assay. The optical density was measured with a microplate reader at 540 nm. The experiment was conducted in triplicate. Data were calculated as percent of cell viability.

#### EGFR and VEGFR-2 Inhibitory Assay

The kinase activity of EGFR was measured by use of BPS Bioscience EGFR kinase assay kit (catalog no. 40321) and the kinase activity of VEGFR-2 was measured by use of HTScan VEGF Receptor 2 kinase assay kit (catalog no. 7788) according to manufacturer's instructions. The results were expressed as IC_50_ and presented in Table 1.

**Table 1:** *In vitro* anticancer activity against HEPG2 cell line and *in vitro* EGFR and VEGFR2 inhibitory activity for the synthesized compounds **1**–**14**.

**Compound**	IC_50_ μM^*^		
	**HEPG2**	**EGFR**	**VEGFR2**
1	0.42	0.14	11.41
2	0.35	0.49	2.12
3	4.07	0.06	0.85
4	0.31	0.13	0.36
5	8.71	0.15	1.12
6	15.73	0.25	1.10
7	1.84	0.25	1.45
8	0.38	0.23	0.46
9	2.30	0.16	0.22
11	0.63	0.30	0.27
12	0.71	0.09	0.23
13	1.81	0.12	0.75
14	6.64	0.11	2.87
15	0.37	0.19	0.37
Erlotinib	10.6	0.13	0.20
Sorafenib	1.06	–	0.03

* *The results given are the mean of three experiments*.

#### Molecular Docking Studies

Molecular Operating Environment (MOE) software model 2014.0901, was used for performing the molecular docking studies. Structures of the synthesized compounds were drawn on the MOE. A Hamiltonian-Force Field-MMFF94x was used to reduce structures' energy. The force field partial charges were calculated for every compound. Default settings were utilized for evaluation of the conformational Stochastic of compounds. The most stable four conformers for each compound have been retained. The X-ray crystal structure of EGFR in complex with tak-285 was downloaded from https://www.rcsb.org/ (PDB ID: 3POZ). The X-ray crystal structure of VEGFR-2 in a complex with sorafenib was downloaded from https://www.rcsb.org/ (PDB ID: 4ASD). The protein-ligand complex bought from the protein data bank was organized for docking: (1) the enzyme was 3D protonated followed by optimization of the system; (2) chain B of the protein along with co-crystallized water molecules were deleted; and (3) determination and isolation of the binding pocket took place, hiding the back bone. MOEDOCK was used to perform the flexible docking of a ligand-rigid receptor for stable conformers. Scoring was performed using the Alpha triangle placement technique and London dG as a function. Force field refinement was applied to the obtained poses through the use of the equal scoring function. Thirty of the most stable docking poses for every ligand were retained with the best scoring conformation was done. Tak-285 was re-docked into the active site of 3POZ and sorafenib into the active site of 4ASD for validation of the docking procedures. The validation results confirmed a near ideal alignment with the original ligands and displayed the same binding interactions as obtained from the X-ray crystallography pdb documents with RMSD of 0.213 and 0.211 Å, respectively, with docking score (S = −18.11 and −17.56 kcal/mol, respectively).

## Results and Discussion

### Chemistry

Multicomponent reactions (MCRs), or one-pot reactions in which at least three different substrates join through the covalent bonds, have been of great interest in synthetic organic chemistry due to the efficiency of giving higher overall yields (Musonda et al., 2004; Dekamin et al., 2013a,b; Dekamin and Eslami, 2014; Rotstein et al., 2014; Sudhapriya et al., 2014; Oshiro et al., 2015; Aly, 2016; Aly et al., 2018). A literature survey reveals that numerous methods were developed for the synthesis of dihydropyrano[2,3-c]pyrazoles (Sudhapriya et al., 2014; Ambethkar et al., 2015; Pati Tripathi et al., 2017). In the beginning, we synthesized the reported pyrano-pyrazole **1** as a staring material by using ethylacetoacetate, hydrazine hydrate, 2-bromobenzaldehyde, and malononitrile, and the reaction was performed in ethanol using triethylamine as basic catalyst (Muramulla and Zhao, 2011).

**Scheme 1:**
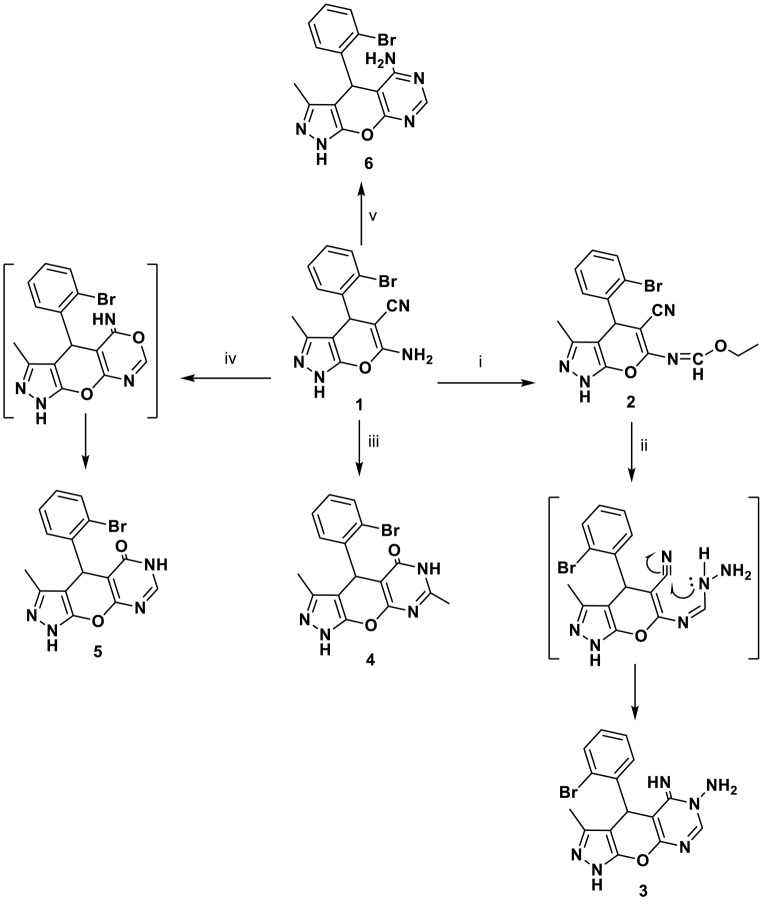
Synthetic pathways for compounds **1**–**6**. ***Reagents and*** ***conditions:* (i)** triethylorthoformate, acetic anhydride, 8 h; **(ii)** NH_2_NH_2_.H_2_O, EtOH, 8 h; **(iii)** acetic anhydride, 10 h; **(iv)** HCOOH, 5 h; **(v)** HCONH_2_, 4 h.

The behavior of o-aminocarbonitrile derivative **1** toward an electrophilic reagent, one-carbon donors, an amide, and an acid was investigated. Thus, the alkylation reaction of the enaminonitrile **1** with triethyl orthoformate in acetic anhydride yielded the ethoxymethylideneamino derivative **2** in a good yield. The reactivity of compound **2** toward hydrazine mono-hydrate was also investigated. Thus, the hydrazinolysis of ethoxymethylideneamino derivative **2** with hydrazine hydrate furnished N-amino-imino derivative **3** through the formation of an intermediate (Scheme 1).

**Scheme 2:**
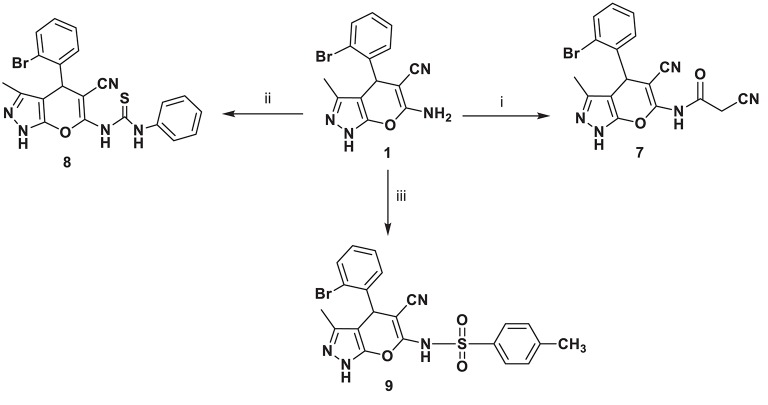
Synthetic pathways for compounds **7**–**9**. ***Reagents and conditions:* (i)** ethyl cyanoacetate, 5 h; **(ii)** phenyl isothiocyanate, EtOH, 6 h; **(iii)** toluene sulfonyl chloride, benzene, pyridine (3 drops), 8 h.

A series of new compounds incorporating pyrimidine moieties attached to pyrano[2,3-*c*]pyrazoles moiety (**4–6**), were synthesized (Scheme 1). Thus, the 4-(2-bromophenyl)-3,7-dimethyl-4,6- dihydropyrazolo[4′, 3′: 5,6]pyrano[2,3-*d*]pyrimidin-5(1*H*)-one **4** was prepared by acetylating amino carbonitrile derivative **1** with acetic anhydride, resulting in an excellent yield. In a similar manner, the behavior of starting material **1** toward the acid derivative was also investigated. Thus, heating compound **1** with formic acid caused cyclization to give the corresponding dihydropyrazolo[4′,3′:5,6]pyrano[2,3-*d*]pyrimidin derivative **5**. Compound **5** was formed via the Dimruth rearrangement illustrated in Scheme 1. Also, pyrano[2,3-*c*]pyrazoles **1** was cyclocondensed with formamide under reflux and afforded the 4-(2-bromophenyl)-3-methyl-1,4-dihydropyrazolo-[4′,3′:5,6]pyrano[2,3-*d*]pyrimidin-5-amine **6**. This work was extended to cover the reactivity of compound **1** toward carbonyl compounds. Thus, condensation of **1** with ethyl cyanoacetate under conditions of reflux gave cyanoacetamide derivative **7** (Scheme 2).

The high electrophilicity and nucleophilicity associated with the carbon and sulfur atoms, respectively, of the isothiocyanates and their extended π electron system make them unique precursors of a large variety of target molecules. Moreover, the reaction of isothiocyanates with amino group of pyrano[2,3-*c*]pyrazoles **1** as nucleophiles was also investigated. Thus, reaction of **1** with phenyl isothiocyanate in boiling ethanol afforded compound **8**.

Sulfonamides are a significant class of compounds in medicinal and pharmaceutical chemistry with several biological applications. In continuation of our interest in biologically active compounds (Alqasoumi et al., 2009; Aly, 2009, 2011; El-Gaby et al., 2018). Refluxing amino pyrano[2, 3-*c*]pyrazole carbonitriles **1** with toluene sulfonyl chloride in benzene containing three drops of pyridine furnished a novel sulfonamide derivative **9** (Scheme 2). Knoevenagel reaction is an important reaction by which the alkenes are obtained from carbonyl compounds and active methylene compounds in the presence of a basic catalyst or Lewis acid catalyst (Aly and Kamal, 2012; Aly et al., 2018). So, through Knoevenagel condensation, the addition of active methylene compound **10** (Yusuf et al., 2017) to the carbonyl group of 2-bromobenzaldehyde in ethanol using piperidine as catalyst occurred, followed by a dehydration reaction in which a molecule of water is eliminated and the product is α, β-unsaturated ketone (a conjugated enone) **11**.

**Scheme 3:**
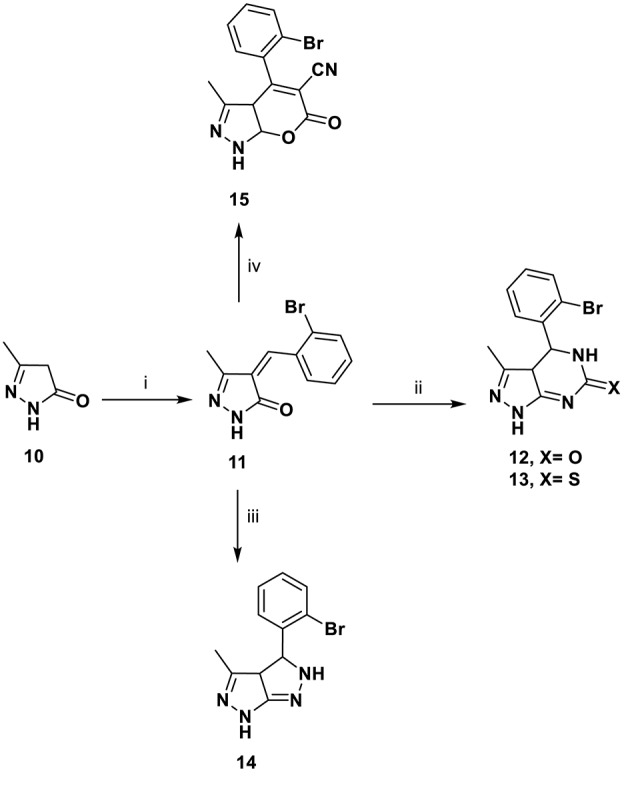
Synthetic pathways for compounds **11**–**15**. ***Reagents and conditions:* (i)** 2-bromobenzaldehyde, EtOH/piperidine (15:1), 5 h; **(ii)** urea or thiourea, glacial acetic acid, 5 h; **(iii)** NH_2_NH_2_.H_2_O, EtOH, 5 h; **(iv)** ethylcyanoacetate, NaOEt, 5 h.

Pyrimidines plays a crucial role in the history of heterocyclic chemistry and used extensively as important pharmacophore in the field of organic chemistry (Aly, 2016; Aly et al., 2018). In our work, pyrazolone **11** was used as a key intermediate for the preparation of several new heterocyclic compounds via its reaction with different nitrogen nucleophiles, such as urea, thiourea, and hydrazine hydrate. Thus, pyrazolo[3,4-*d*]pyrimidine derivatives **12** and **13** were synthesized by the condensation of *(E*)-4-(2-Bromobenzylidene)-5-methyl-2,4-dihydro-3*H*-pyrazol-3-one **11**, urea, and/or thiourea in glacial acetic acid via β-attack on the C = C moiety in compound **11**, followed by 1,6- intramolecular dipolar cyclization. Similarly, the reaction of compound **11** with hydrazine hydrate gave pyrazolo[3,4-*c*]pyrazole **14** via β-attack on C = C moiety in compound **11** followed by 1,5- dipolar cyclization. The reaction of ethyl cyanoacetate in the presence of sodium ethoxide gave the dihydropyrano[2,3-*c*]pyrazole-5-carbonitrile **15** (Scheme 3). The structures of the target compounds were proven by elemental and spectral data and were consistent with assigned structures as presented in details in the experimental section.

### *In vitro* Biological Evaluation

#### *In vitro* Anticancer Activity

The newly synthesized compounds were evaluated for their *in vitro* anticancer activity against HEPG2 human tumor cell line and compared to erlotinib and sorafenib as reference drugs through the use of an MTT assay. The results were displayed as IC_50_, which is the concentration that kills 50% of the cells as shown in Table 1. According to the results obtained, all the tested compounds except compound **6** exhibited significant results more potent than the reference drugs. Compounds **1**, **2**, **4**, **8**, **11**, **12**, and **15** showed nearly 10 fold more activity than erlotinib (10.6 μM), and they were also more potent than sorafenib (1.06 μM) with IC_50_ ranging from 0.31 to 0.71 μM. Slightly lower activity was observed for compounds **7**, **9**, and **13** (1.84, 2.30, and 1.81 μM, respectively), and compounds **3**, **5**, and **14** with much lower activities (4.07, 8.71, and 6.64 μM, respectively).

By observing the SAR of the compounds, we can make several conclusions:

- Introducing different substituents to the 6-NH_2_ group greatly influenced the activity in the dihydropyrano-pyrazole derivatives. The starting compound **1** showed significant activity (0.42 μM), which is ameliorated upon introducing the ethyl formamidate group in compound **2** (0.35 μM) and phenyl thiourea moiety in compound **8** (0.38 μM). While, the presence of cyanoacetamide and sulfonamide moieties in compounds **7** and **9** resulted in slight decrease in the activity (1.84 and 2.30 μM). This activity was restored due to substitution of 6-NH_2_ with a carbonyl group as in compound **15** (0.37 μM).
- Concerning the pyrazolo-pyrano-pyrimidine derivatives **3**–**6**, the type and position of the substituent on the pyrimidine ring greatly affected the activity. The most potent among these compounds was **4** (0.31 μM), having 5-one 7-methyl substituents on pyrimidine ring, and it is the most potent compound in this study. A drop in the activity was observed for the 5-imino 6-amino derivative **3** (4.07 μM), 5-one derivative **5** (8.71 μM), and the 5-amino derivative **6** (15.73 μM) underlining the importance of aliphatic substitution on position 7 of the pyrimidine ring for the activity.
- The pyrazole derivative **11** showed a significantly high activity (0.63 μM), which is slightly decreased upon cyclization to pyrazolopyrimidine derivatives **12** and **13** (0.71 and 1.81 μM, respectively). Meanwhile, a 6 folds decrease in activity was obtained upon adding an additional pyrazole ring, as in compound **14** (6.64 μM).
- Generally, we can observe that, for the bicyclic compounds, substitution on position 6 was important for activity, while, for the tricyclic compounds, substitution on position 7 resulted in the most potent compounds.

#### *In vitro* EGFR and VEGFR-2 Inhibitory Assays

All the synthesized compounds were evaluated for their *in vitro* EGFR and VEGFR-2 inhibitory activity, and the IC_50_ values were calculated and compared to the reference drug erlotinib.

By examining the structures and EGFR inhibitory activity of the tested compounds, the following SAR could be concluded:

- Four compounds showed higher activity than erlotinib (0.13 μM), the pyrano-pyrazolo-pyrimidine derivative **3** (0.06 μM). This may be attributed to the presence of 5-imino 6-amino groups providing additional hydrogen bonding within the active site. The two pyrazolopyrimidine derivatives **12** and **13** showed high inhibitory activity (0.09 and 0.12 μM, respectively), but the 6-substitution on the pyrimidine ring with carbonyl group in compound **12** was better than the thione in **13**.
- Compounds **1**, **4**, **5**, **9**, **14**, **and 15** showed comparable activity to erlotinib, while compounds **2**, **6**, **7, 8**, and **10** showed 2–3 fold lower activity than the reference drug.

The VEGFR2 inhibitory activity for the tested compounds was evaluated and the following SAR was observed:

- Three compounds were equipotent compared to erlotinib (0.20 μM). The sulfonamide derivative **9** (0.21 μM), having *p*-methyl substituent on the terminal aromatic ring, might provide additional hydrophobic interaction within the active site. The pyrazole derivative **11** (0.27 μM) containing a free carbonyl group was able to provide H-bonding within the receptor. Pyrazolopyrimidine derivative **12** (0.23 μM), containing a free carbonyl and additional H-bond donor NH were the most potent compounds in this study, while none of the compounds were as potent as sorafenib (0.03 μM).
- The pyrazolo-pyrano-pyrimidine derivative **4** (having a carbonyl and a free methyl group on the pyrimidine ring), the thioureido-pyranopyrazole derivative **8**, and the 6-oxo pyranopyrazole derivative **15** showed moderate activity that was slightly lower than erlotinib.
- Compounds **2**, **5**–**7**, **13**, and **14** showed 1–2 fold lower activity than erlotinib, and, finally, the starting compound **1** was found to have an exceptional low activity on VEGFR-2 (11.41 μM).

Examining the structures of the most potent compounds demonstrated the following SAR as dual EGFR and VEGFR-2 inhibitors:

- The presence of H-bond donors 5-imino 6-amino on the pyrimidine ring in compound **3** had a great impact on EGFR inhibition with moderate VEGFR-2 inhibition.
- The introduction of a sulfonamide group with p-methyl moiety on the terminal aromatic ring in compound **9** resulted in dual EGFR and VEGFR-2 inhibition.
- The free pyrazolone ring in compound **11** resulted in better VEGFR2 inhibition. Upon cyclization to pyrazolopyrimidine, 6-substitution on pyrimidine ring greatly affected the activity. 6-one derivative **12** showed dual EGFR and VEGFR-2 inhibition, but the inhibitory activity on VEGFR-2 was significantly decreased in 6-thioxo derivative **13**. The introduction of another pyrazole ring in compound **14** resulted in a drop in VEGFR-2 inhibition with better activity on EGFR.

#### Molecular Docking Studies

Docking studies was carried out for compounds **9** and **12**, which showed excellent dual EGFR and VEGFR-2 inhibition to illustrate the molecular reasons for the observed inhibitory activity.

**Figure 2:**
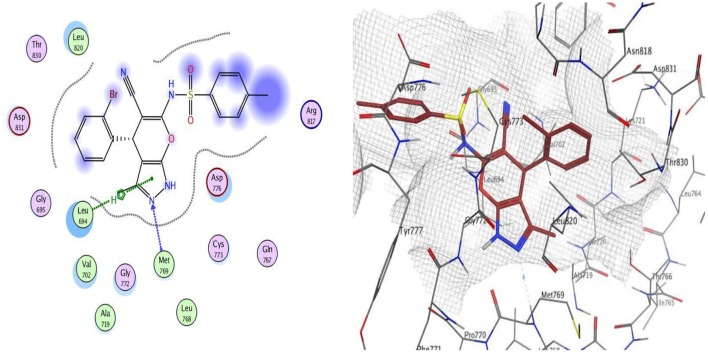
2D and 3D interaction map of compound **9** within the active site of EGFR (3POZ).

**Figure 3:**
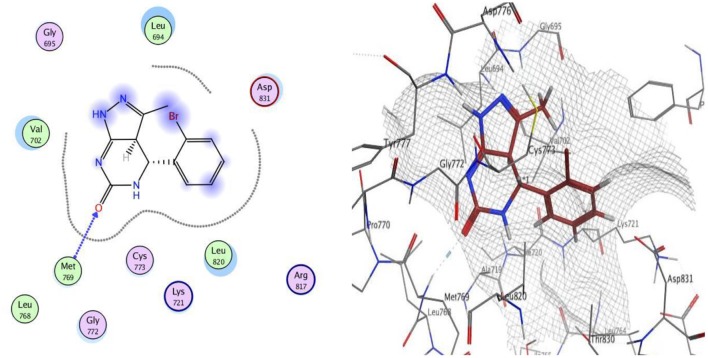
2D and 3D interaction map of compound **12** within the active site of EGFR (3POZ).

The docking of compounds **9** and **12** at the EGFR active site showed that the two compounds bind with the essential amino acids, and compound **9** H-bonds with Met769 through N of pyrazole ring with a bond length equal to 1.93 Å and with Leu694 with bond length 1.65 Å. Meanwhile, compound **12** interacts within the active site by one hydrogen bond with Met769 through carbonyl group (1.03 Å). The greater EGFR inhibitory activity observed for compound **12** over compound **9** could be interpreted to be attributed to the smaller bond length between the interacting groups and the amino acids, which leads the compound into pushed deeper inside the active site, resulting in better inhibitory activity. Moreover, compound **12** showed a better docking score (−12.90 Kcal/mol) than compound 9 (−12.01 Kcal/mol) (Figures 2, 3).

**Figure 4:**
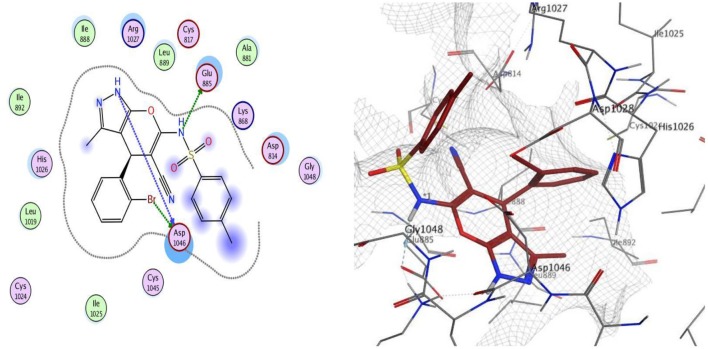
2D and 3D interaction map of compound **9** within the active site of VEGFR2 (4ASD).

**Figure 5:**
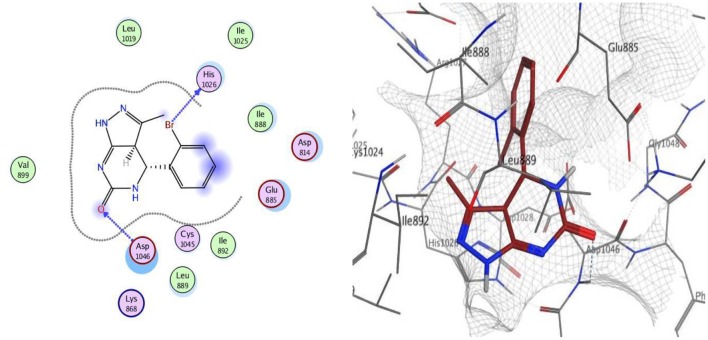
2D and 3D interaction map of compound **12** within the active site of VEGFR2 (4ASD).

Docking was performed for compounds **9** and **12** within VEGFR-2 active, and the results elucidated that compound **9** was able to interact with Asp1046 through H-bonding with the NH of the pyrazole ring (1.65 Å) and also with a bromine atom (1.79 Å), and, together with another H-bond interaction with Glu885 through the NH of sulfonamide moiety, these findings are supported by previous literature, correlating potent inhibitory activity to the interaction with these two amino acids. Compound **12** interacted within the active site with two H-bonds with Asp1046 and His1026 through carbonyl group (1.70 Å) and bromine atom (1.62 Å), respectively. The good VEGFR-2 inhibitory activity of compounds **9** and **12** were illustrated by their good docking scores (−11.88 and −11.61 Kcal/mol) (Figures 4, 5).

## Conclusion

A series of novel fused pyrazole derivatives were synthesized and evaluated for their *in vitro* anticancer activity against the HEPG2 human cancer cell line, and they were compared to erlotinib and sorafenib as reference drugs. Seven compounds, **1**, **2**, **4**, **8**, **11**, **12**, and **15**, showed nearly 10 fold more activity than erlotinib (10.6 μM), with IC_50_ ranging from 0.31 to 0.71 μM. *In vitro* EGFR and VEGFR-2 inhibitory activity were performed for the synthesized compounds, and the results identified compound **3** as the most potent EGFR inhibitor (IC_50_ = 0.06 μM) and compound **9** as the most potent VEGFR-2 inhibitor (IC_50_ = 0.22 μM). Moreover, compounds **9** and **12** revealed potent dual EGFR and VEGFR-2 inhibition, and compound **12** was the best compound; both *in vitro* anticancer activity against the HepG2 cell line and *in vitro* EGFR and VEGFR2 inhibitory activity. These results were supported by docking studies of these two compounds within the active sites of both enzymes to better understand their mode of action that led to good inhibitory activity. Docking results showed that compounds **9** and **12** interacted with the key amino acids responsible for activity in both enzymes. These preliminary results introduce novel moieties as dihydro-pyrano-pyrazole and pyrazolo-pyrimidine derivatives as promising scaffolds to produce potent EGFR and VEGFR-2 inhibitors.

## Data Availability Statement

The raw data supporting the conclusions of this article will be made available by the authors, without undue reservation, to any qualified researcher.

## Author Contributions

NS, ME-G, and HA contributed conception and design of the study. RO synthesized the target compounds. ME-G performed docking study and wrote the first draft of the manuscript. NS, HA, and RO wrote chemistry experimental section of the manuscript. All authors contributed to manuscript revision and read and approved the submitted version.

### Conflict of Interest

The authors declare that the research was conducted in the absence of any commercial or financial relationships that could be construed as a potential conflict of interest.
